# Development of a Novel Mitochondrial Dysfunction-Related Alzheimer’s Disease Diagnostic Model Using Bioinformatics and Machine Learning

**DOI:** 10.2174/0115672050353736241218054012

**Published:** 2024-12-26

**Authors:** Kuo Zhang, Kai Yang, Gongchang Yu, Bin Shi

**Affiliations:** 1 Neck-Pain Hospital of Shoulder and Lumbocrural Shandong First Medical University, Shandong First Medical University & Shandong Academy of Medical Sciences, Jinan, China;; 2 Department of Neurology, Shandong University of Traditional Chinese Medicine, Jinan, China

**Keywords:** Machine learning, devastating disease, Alzheimer’s disease, CytoHubba algorithm, mitochondrial genes, kynurenic acid

## Abstract

**Introduction:**

Alzheimer’s disease (AD) represents the most common neurodegenerative disorder, characterized by progressive cognitive decline and memory loss. Despite the recognition of mitochondrial dysfunction as a critical factor in the pathogenesis of AD, the specific molecular mechanisms remain largely undefined.

**Methods:**

This study aimed to identify novel biomarkers and therapeutic strategies associated with mitochondrial dysfunction in AD by employing bioinformatics combined with machine learning methodologies. We performed Weighted Gene Co-expression Network Analysis (WGCNA) utilizing gene expression data from the NCBI Gene Expression Omnibus (GEO) database and isolated mitochondria-related genes through the MitoCarta3.0 database. By intersecting WGCNA-derived module genes with identified mitochondrial genes, we compiled a list of 60 mitochondrial dysfunction-related genes (MRGs) significantly enriched in pathways pertinent to mitochondrial function, such as the citrate cycle and oxidative phosphorylation.

**Results:**

Employing machine learning techniques, including random forest and LASSO, along with the CytoHubba algorithm, we identified key genes with strong diagnostic potential, such as ACO2, CS, MRPS27, SDHA, SLC25A20, and SYNJ2BP, verified through ROC analysis. Furthermore, an interaction network involving miRNA-MRGs-transcription factors and a protein-drug interaction network revealed potential therapeutic compounds such as Congo red and kynurenic acid that target MRGs.

**Conclusion:**

These findings delineate the intricate role of mitochondrial dysfunction in AD and highlight promising avenues for further exploration of biomarkers and therapeutic interventions in this devastating disease.

## 1 INTRODUCTION

Alzheimer’s disease (AD) is a progressive neurodegenerative disorder that represents the primary cause of dementia, accounting for 60-80% of all dementia cases [1, 2]. Alzheimer’s disease is characterized by a steady deterioration in memory and cognitive ability, and roughly 43% of patients require significant nursing care [3]. Given the irreversible nature of its pathological process and the lack of effective early diagnostic and clinical management strategies, AD has emerged as a pressing research focus within the field of neurodegenerative diseases. The neuropathological hallmarks of AD include the extracellular accumulation of insoluble beta-amyloid protein (Aβ) peptides and the formation of neurofibrillary tangles (NFTs) composed of hyperphosphorylated tau protein [4, 5]; however, targeted treatments addressing these features have shown limited efficacy. For instance, numerous phase III clinical trials have demonstrated that Aβ inhibitors do not significantly enhance cognitive function in AD patients [6-8]. Despite significant advancements in alleviating AD symptoms [9-11], further research is imperative to achieve early prevention and intervention through the modulation of mitochondrial dysfunction. Understanding the diverse molecular mechanisms driving AD progression and identifying potential biomarkers is crucial for establishing a foundational understanding of early disease diagnosis and clinical treatment strategies.

Mitochondria, vital for aerobic respiration and energy production in eukaryotic cells, regulate metabolic homeostasis, intracellular signaling, and cell apoptosis, thereby maintaining cellular physiological function [12]. Neurons exhibit the highest energy consumption rate among cell types, primarily to sustain the ion gradient necessary for electrophysiological activity, neurotransmission, and short-term synaptic plasticity [13, 14]. The energy required for these processes relies heavily on mitochondrial supply, making the brain particularly susceptible to mitochondrial dysfunction [15].

Mitochondrial dysfunction leads to oxidative damage to mitochondrial components, decreased adenosine triphosphate (ATP) synthesis, and subsequent impairment of synaptic plasticity, manifesting as learning and memory deficits [16]. Extensive research has demonstrated a wide range of mitochondrial abnormalities in the brains of AD patients, including altered mitochondrial enzyme activity, defective mitochondrial quality control, and oxidative damage [17-19]. Mitochondrial dysfunction and metabolic abnormalities occur in the early stages of AD progression, with systemic mitochondrial defects observed in AD patients compared to controls [20]. This suggests that mitochondrial dysfunction is a critical component of the pathogenic cascade in AD.

Despite recognizing mitochondrial dysfunction as a pivotal pathological mechanism in AD onset and progression, identifying definitive biomarkers for early diagnosis remains a challenge. Therefore, utilizing bioinformatics analysis to elucidate the connection between genes associated with mitochondrial dysfunction and AD and identifying potential biomarkers for early AD detection and corresponding therapeutic interventions holds significant promise.

## 2 METHODS

### 2.1 Data Source and Preprocessing

We utilized brain tissue chip data from the GSE132903 dataset [21] on the GPL10558 platform of the Gene Expression Omnibus (GEO) database. This dataset comprises data from 97 Alzheimer’s Disease (AD) patients and 98 normal controls. Additionally, we selected the GSE5281 dataset [22, 23], which has a larger sample size and includes 161 samples, for validation [24, 25]. The GSE5281 dataset (GPL570) extracted postmortem brain tissue samples from 87 AD patients and 74 controls. The sample tissues in both datasets were analyzed using Illumina Human HT-12 v4 and Affymetrix Human Genome U133 Plus 2.0 Array, respectively (Table **1**). The raw data from the GSE132903 and GSE5281 datasets underwent R software processing. The limma package [26] and affy package [27] were used to normalize the raw matrix. Besides, we downloaded 1136 human mitochondrial proteins and pathway-related genes from the MitoCarta database to identify Mitochondrial Dysfunction-related genes (MRGs). Fig. (**1**) illustrates the flowchart of this study.

### 2.2 Weighted Gene Co-expression Network Analysis (WGCNA)

WGCNA analyzed the dataset GSE132903 to uncover the link and mechanism between gene networks and AD. Following the scale-free topology criterion, an appropriate soft-thresholding power was chosen to calculate and convert the correlation coefficients among genes into an adjacency matrix, effectively indicating the strength of connections between genes. The soft-thresholding power was selected to achieve a scale-free network with an r^2^ value of 0.9. Subsequently, the adjacency matrix was further transformed into a topological overlap matrix, which captured the topological similarity between genes. Clusters were formed using the k-means clustering method to group genes that displayed analogous expression patterns. The gray module indicated genes that did not belong to any module.

### 2.3 Functional Enrichment Analysis

Functional enrichment analysis was carried out in three Gene Ontology (GO) domains: biological process (BP), cellular component (CC), and molecular function (MF). The Kyoto Encyclopedia of Genes and Genomes (KEGG) database was utilized for pathway datasets encompassing biological functions, diseases, chemicals, and drugs. The enrichment analysis was performed using the clusterProfiler R package to identify the biological functions of genes and their associated pathways [28]. Significant enrichment was determined based on *P*-values < 0.05.

### 2.4 Identification of Critical Genes using Machine Learning

The chosen MRGs were subjected to analysis using three machine learning algorithms: random forest (RF), least absolute shrinkage and selection operator (LASSO), and support vector machine (SVM). The randomForest package was used to sequence the genes according to the variable importance of the training set. LASSO logistic regression analysis was conducted using the glmnet package, setting the response type to binomial and alpha to 1. SVM analysis was undertaken to identify a hyperplane for classifying the data points, followed by the subsequent screening of critical genes.

In our LASSO regression analysis, to mitigate overfitting effectively and ascertain the optimal λ value, we employed a cross-validation strategy facilitated by the cv.glmnet function. This function automatically partitions the dataset into ten subsets by default, executing a k-fold cross-validation with k set to 10. In each iteration, a subset of the data is designated as the validation set, while the remaining data serves as the training set. This methodology not only bolsters efficiency but also guarantees robust model performance on unobserved data, thereby forestalling overfitting.

For the Support Vector Machine (SVM) analysis, we similarly adopted a 10-fold cross-validation approach. The dataset is evenly split into ten segments, with nine segments dedicated to training the SVM model and the remaining segment used for validation in each iteration. Despite the absence of explicit labeling as “training set” and “validation set,” the core principle of k-fold cross-validation inherently provides this distinction, rendering it a ubiquitous technique for assessing and selecting the performance of machine learning models.

In our random forest analysis, we distinctly differentiated between the training set and the test set. By utilizing random sampling and setting a seed (set.seed(2023)) for reproducibility, we leveraged the sample function to generate indices that partitioned the clinical and expression data into training and test sets. During the training phase, the random forest model was exclusively constructed using the training set. In the validation phase, both the training set (for internal evaluation) and the test set (for external evaluation) were employed to conduct a comprehensive assessment of the model’s performance, ensuring the objectivity and precision of our evaluation results.

The genes obtained through the three machine learning algorithms were intercrossed and merged, and VennDiagram [29] was used to visualize the intersection of gene lists.

The online database STRING (http://stringdb.org) was utilized to investigate the interactions among the proteins encoded by the MRGs. The PPI network was constructed through the utilization of the software Cytoscape [30]. To detect densely connected regions and modules within the PPI network, the CytoHubba [31] plug-in was employed. The CytoHubba algorithm exhibits significant advantages in terms of interpretability, comprehensiveness, and computational efficiency. It overcomes the lack of interpretability in machine learning methods and the inadequacy of traditional statistical methods in neglecting the complex relationships within biological networks. Six strategies were used to screen and ascertain the top 10 hub genes. The nodes with the highest scores based on Betweenness, Closeness, Degree, EPC, MNC, and MCC were selected and recognized as hub genes. After performing the intersection and merging of gene lists obtained from the six algorithms, the overlapping genes were visualized using the Venn diagram tool to illustrate their commonality.

### 2.5 Integration of Key Genes Obtained through Machine Learning and CytoHubba Algorithm

We employed PPI network analysis and machine learning models to obtain the critical genes with diagnostic value in MRGs. However, we observed a limited overlap between the critical genes obtained from these two methods. Firstly, machine learning methods utilize feature selection and model training to discover potential essential genes from large-scale gene expression data, even if these genes may not interact directly in the PPI network. Secondly, PPI network analysis focuses on the interactions between genes, revealing their essential roles in cellular processes and signal transduction. Therefore, we decided to merge the critical genes obtained from both methods to obtain more comprehensive and reliable results. This approach provides a more extensive collection of crucial genes, encompassing important information revealed by both methods. To explore potential connections between critical genes, we used ggcorplot to create a correlation heatmap illustrating their relationships. The ggplot2 package was used to visualize the expression of critical genes between the AD and control groups. Based on the levels of expression in the GSE132903 and GSE5281 datasets, genes without significantly different expression were excluded.

### 2.6 Construction and Validation of Diagnostic Models

The ROC (Receiver Operating Characteristic) curve serves as an effective tool for intuitively reflecting the performance of diagnostic models, enabling researchers to quickly grasp the sensitivity and specificity of the models, thereby comprehensively assessing their diagnostic capabilities. The Area Under the Curve (AUC) of the ROC is a crucial quantitative indicator for evaluating model quality, with an AUC value closer to 1 indicating stronger predictive power of the model. By calculating the AUC value, we can objectively and quantitatively compare the diagnostic performance of different models, further screening out the optimal diagnostic model. In the GSE132903 dataset, we conducted ROC curve analysis on key MRGs using the ROCR software package [32]. Meanwhile, in the external dataset GSE5281, we employed ROC univariate analysis to validate the sensitivity and specificity of the diagnostic model and assessed the predictive significance of the key MRGs. Furthermore, a nomogram was developed to forecast the progression of AD. By drawing a line between the different axes to connect the scores for each variable, the scores for each variable are added up to a total score, thus allowing the probability of the outcome occurring to be predicted. A calibration curve is employed to assess the predictive accuracy of the diagnostic model.

### 2.7 Analysis of Immune Cell Infiltration

The CIBERSORT algorithm was applied to determine the relative proportions of immune cell types in each sample [33]. The analysis was performed using the CIBERSORT and parallel packages, along with e1071 and preprocessCore in R. To accentuate the disparities in immune infiltration between AD and control samples, we used violin plots generated with the ggplot2 package. A significance level of *P<*0.05 was employed as the threshold for statistical significance.

### 2.8 Correlation Analysis between Immune Cell Subsets and Critical Biomarkers of MRGs

We conducted Spearman correlation analysis using R packages ggstatsplot to assess the correlation between critical diagnostic biomarkers of AD and immune cell subsets. The R packages ggsci, ggplot2, and tidyverse were utilized to visualize the results. We used a cut-off value of *P<*0.05.

### 2.9 Construction of miRNA-MRGs-transcription Factor (TF) Interaction Network

TFs and microRNAs (miRNAs) individually or in concert control gene interaction networks. TFs are a type of protein that regulates mRNA transcription by binding to specific DNA sequences. Additionally, miRNAs are short, non-coding RNAs, approximately 22 nucleotides in length, that inhibit translation and promote mRNA degradation by binding to target mRNAs. The TF-MRGs and MRGs-miRNA interaction data were obtained from JASPAR and miRTarBase, respectively. NetworkAnalyst 3.0 (https://www.networkanalyst.ca/) was used to analyze and generate the TF-MRGs-miRNA interaction network. The results were visualized using Cytoscape software to establish the gene interaction network.

### 2.10 Construction of Protein-drug Interaction Network

This study aims to predict protein-drug interactions to elucidate the mechanism of drug action and provide clinical guidance. We used Enrichr (https://maayanlab.cloud/Enrichr/) with the Drug Signatures database (DSigDB) to identify drug molecules [34]. The DSigDB database integrates drug-gene interaction data from various sources, including experimental data, literature data, and public database resources, to construct a more comprehensive and accurate drug-gene interaction network. Additionally, DSigDB employs machine learning algorithms and other methodologies to build predictive models. These models are capable of predicting novel drug-gene interactions, providing new leads and insights for drug discovery and development.

## 3 RESULTS

### 3.1 Data Source and Preprocessing and WGCNA

**Fig. (2):**
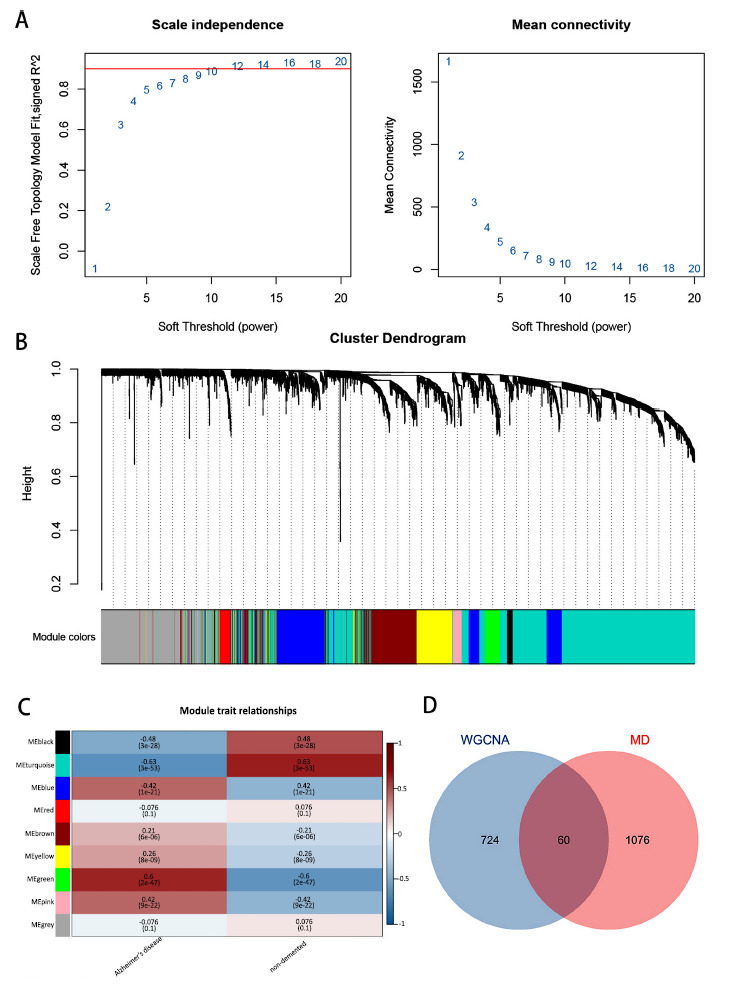
The investigation of module genes in GSE132903 was conducted using Weighted gene co-expression network analysis. (**A**) Soft Threshold power determination for GSE132903; (**B**) Display of origin and merged modules under the clustering tree for GSE132903; (**C**) Correlation heatmap between module genes and AD patients; (**D**) Venn diagram of overlapping module genes and mitochondria-related genes.

To explore the correlation between clinical features and genes, we constructed a gene co-expression network using the GSE132903 dataset. Non-compliant samples were excluded, and a scale-free topology model was established by calculating the soft threshold using the conventional approach. The optimal soft threshold was determined to be 12 (Fig. **2A**). Following weighted filtering, nine modules were identified within the network (Fig. **2B**). Subsequently, we calculated the correlation between these modules and patients with Alzheimer’s disease (AD) compared to the control group. Fig. (**2C**) revealed that the green module, comprising 724 genes, exhibited the strongest positive correlation with AD (r=0.6). Conversely, the turquoise and black modules showed the most significant negative correlations with AD (r=0.63 and 0.48, respectively). We selected the green module for overlap analysis with genes from the MitoCarta database (Fig. **2D**). Ultimately, 60 genes from the module overlapped with genes in the MitoCarta database, identifying them as candidate genes for further investigation.

### 3.2 Functional Enrichment Analysis

**Fig. (3):**
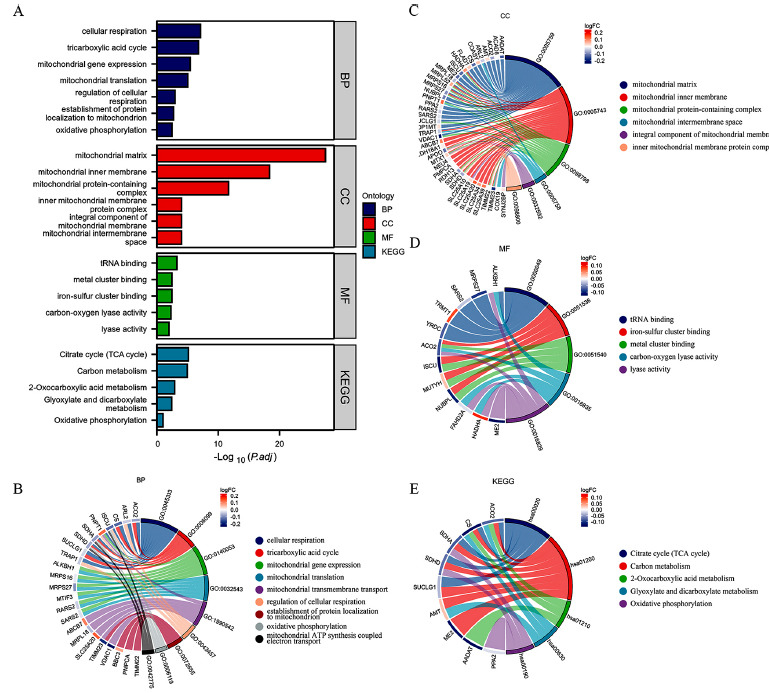
Functional enrichment analysis of critical genes. (**A**) Ranking of adjusted P-values of items; (**B**) BP enrichment results; (**C**) CC enrichment results; (**D**) MF enrichment results; (**E**) Enrichment pathways from KEGG for MRGs.

To elucidate the functions of mitochondria-related genes (MRGs) associated with AD, we conducted Gene Ontology (GO) and Kyoto Encyclopedia of Genes and Genomes (KEGG) pathway enrichment analyses (Fig. **3A**). GO enrichment analysis indicated that MRGs were predominantly enriched in processes such as mitochondrial gene expression, mitochondrial translation, inner mitochondrial membrane protein complex assembly, and mitochondrial protein-containing complex assembly (Figs. **3B-D**). KEGG pathway enrichment analysis showed that MRGs were mainly enriched in pathways including the Citrate cycle (TCA cycle), Carbon metabolism, 2-Oxocarboxylic acid metabolism, Glyoxylate and dicarboxylate metabolism, and Oxidative phosphorylation (OXPHOS) (Fig. **3E**). Notably, both the TCA cycle and OXPHOS were found in both GO and KEGG enrichment analyses, suggesting that altered molecular expression levels in these pathways may be a contributing factor to mitochondrial dysfunction in AD.

### 3.3 Identification of Critical Genes Using Machine Learning

**Fig. (4):**
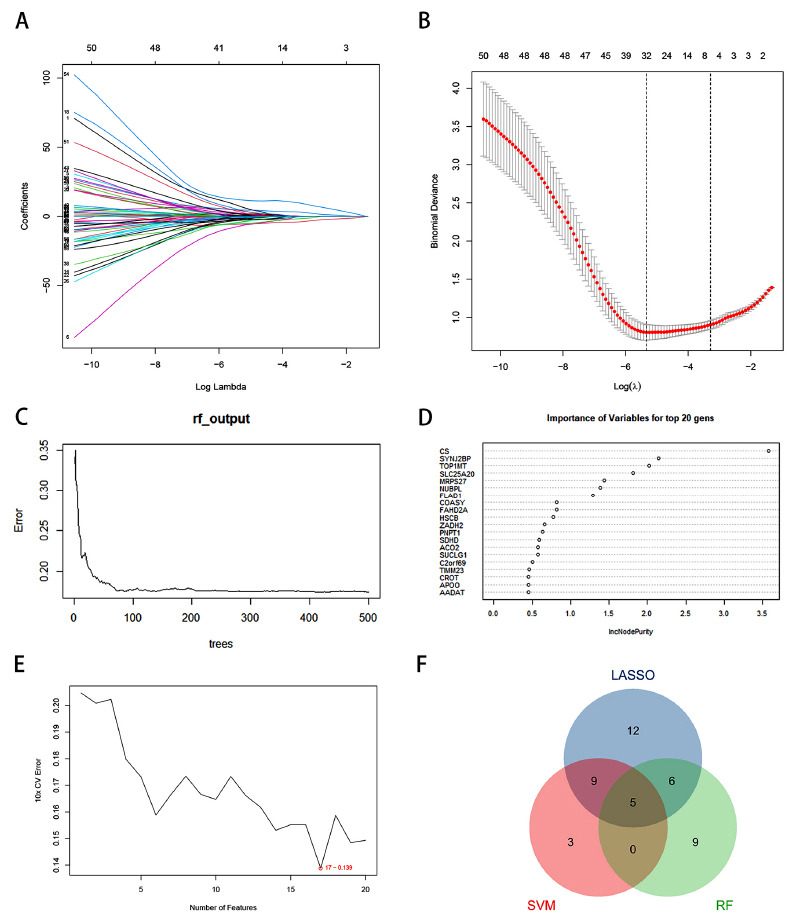
The process of identifying feature MRGs utilizing machine learning algorithms. (**A**) Determination of the optimal parameter (lambda) in the LASSO model; (**B**) Distribution of coefficients for the 32 most correlated MRGs based on the optimal lambda; (**C**) The randomForest algorithm is used to construct a diagnostic model; (**D**) Ranking of 20 genes screened according to the Importance of Variables; (**E**) Selection of the most relevant MRGs using the SVM algorithm; (**F**) Venn diagram showing the MRGs filtered by the three machine learning algorithms.

To identify critical genes, we employed Random Forest (RF), LASSO regression, and Support Vector Machine (SVM) algorithms to construct prediction models. LASSO regression results identified 32 genes as most associated with AD (Figs. **4A**, **B**). According to the RF algorithm, the top 20 genes were selected as critical genes based on their variable importance (Figs. **4C**, **D**). The SVM algorithm identified 17 key genes (Fig. **4E**). By intersecting the genes identified by these three machine-learning methods, we obtained five genes: MRPS27, FAHD2A, TOP1MT, SLC25A20, and SYNJ2BP (Fig. **4F**).

### 3.4 Construction and Analysis of PPI Network

**Fig. (5):**
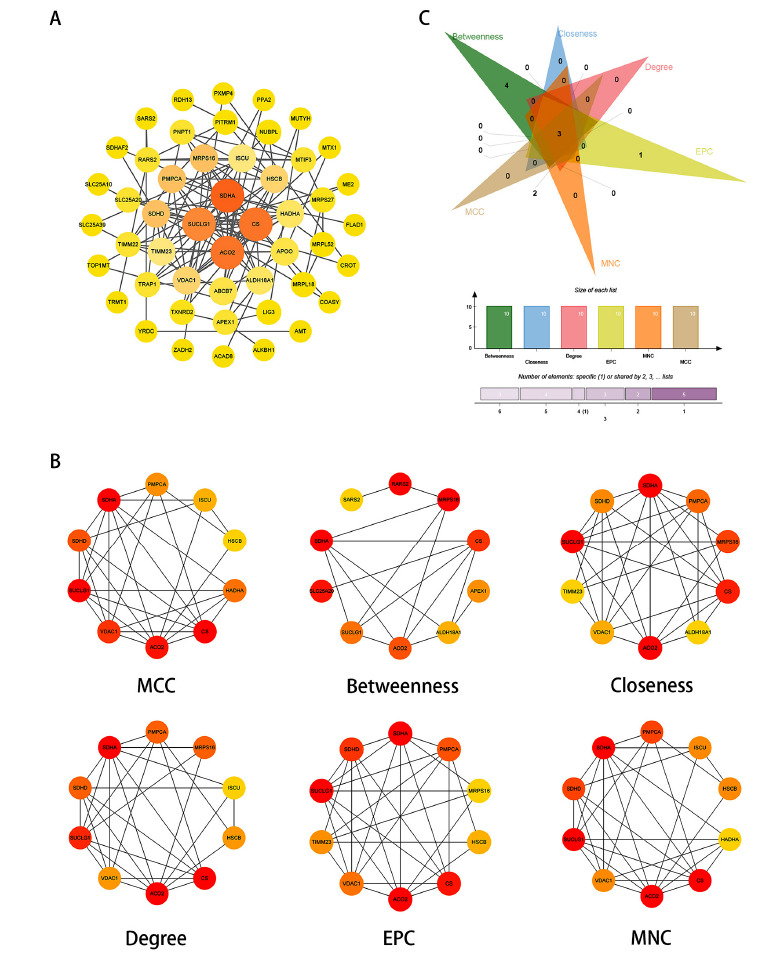
Protein-protein interaction network of MRGs and screening of hub genes. (**A**) Protein-protein interaction network constructed by MRGs using Cytoscape; (**B**) Six CytoHubba algorithms to obtain the network module; (**C**) The Venn diagram of the genes selected by six algorithms.

To investigate the interactions between MRGs and their corresponding proteins, we constructed a Protein-Protein Interaction (PPI) network using the STRING database, which included 60 MRGs. The network consisted of 118 edges and 60 nodes (Fig. **5A**). Using the CytoHubba plugin in Cytoscape, we applied six selection methods to identify prominent modules within the PPI network (Fig. **5B**). By overlapping the results obtained from these different methods, we successfully identified hub genes within the PPI network. These hub genes, namely SDHA, CS, and ACO2, are pivotal in the PPI network and hold substantial functional implications in the pathogenesis of AD (Fig. **5C**).

#### 3.4.1 Integration of Key Genes Derived from Machine Learning and CytoHubba Algorithm

**Fig. (6):**
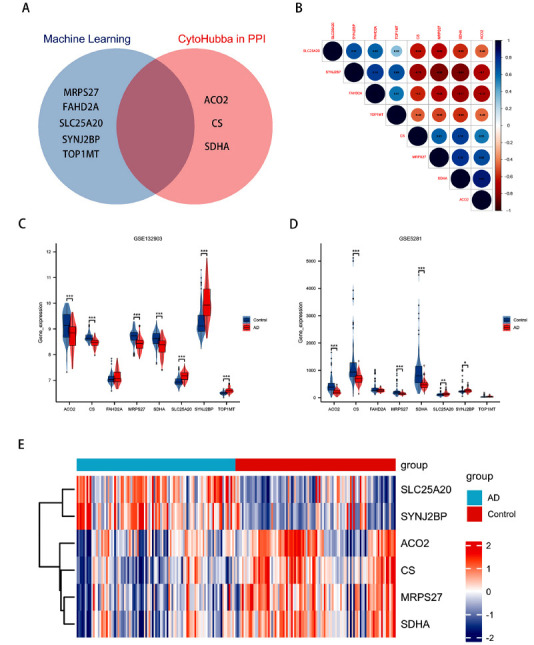
Key genes were screened according to their expression differences. (**A**) Key MRGs obtained by combining machine learning and CytoHubba algorithms; (**B**) Heat maps of correlations between 8 key MRGs; (**C**) Violin diagram of critical MRGs expression in GSE132903; (**D**) Violin diagram of the expression of key MRGs in GES5281; (**E**) Heat maps of expression levels of 6 critical MRGs in AD.

Eight genes, identified through the combination of protein-protein interaction (PPI) networks and machine learning algorithms, were integrated (Fig. **6A**). The correlation heatmap revealed a tight interrelation among these mitochondrial-related genes (MRGs) (Fig. **6B**). Analysis of their differential expression in the validation set indicated that the expression variations of two genes, FAHD2A and TOP1MT, were insufficient for validation (Figs. **6C**, **D**). Consequently, these two genes were excluded, leaving six critical genes with diagnostic significance for Alzheimer’s disease (AD): ACO2, CS, MRPS27, SDHA, SLC25A20, and SYNJ2BP (Fig. **6E**, Table **2**).

#### 3.4.2 Construction and Validation of Diagnostic Models

**Fig. (7):**
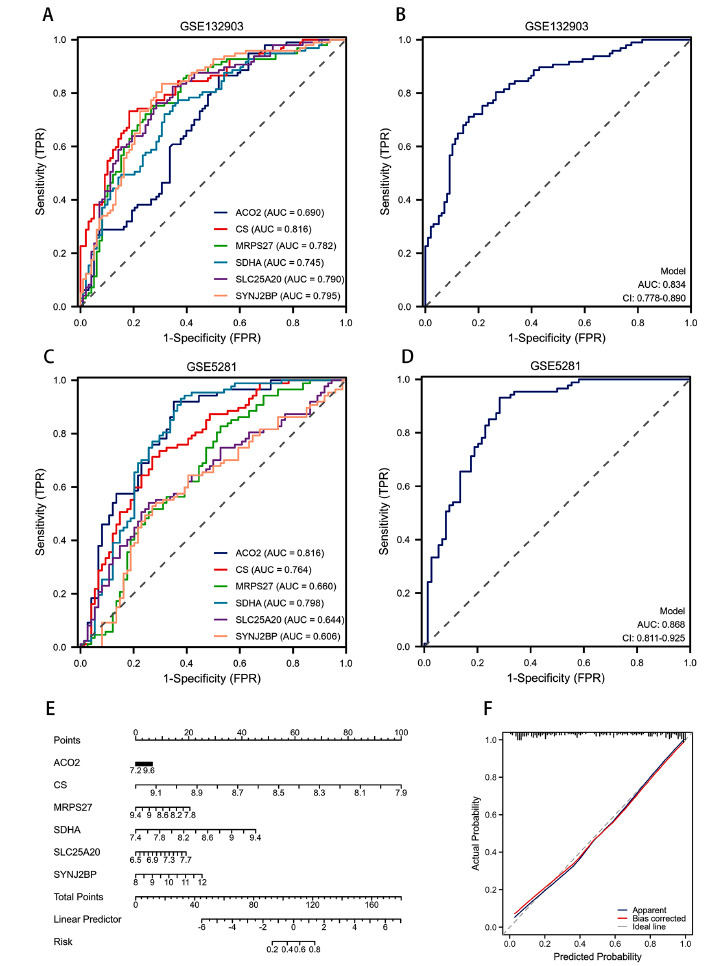
Verification of diagnostic markers. (**A, B**) The ROC curve of the diagnostic efficacy in training set GSE132903; (**C, D**) The ROC curve demonstrating the diagnostic efficacy in verification set GSE5281; (**E**) Construction of a nomogram model based on 6 key MRGs to predict the risk of AD patients; (**F**) The calibration curve of the diagnostic model was constructed to verify the predictive performance.

The dataset GSE132903 was utilized for ROC curve analysis to evaluate the predictive power of the key MRGs. The results demonstrated that CS exhibited the highest Area Under the Curve (AUC) value of 0.816, followed by SYNJ2BP (0.795), SLC25A20 (0.790), MRPS27 (0.782), SDHA (0.745), and ACO2 (0.690) (Fig. **7A**). Subsequently, an external dataset, GSE5281, was introduced to verify the model’s accuracy and sensitivity, yielding AUC values of 0.816 for ACO2, 0.798 for SDHA, 0.764 for CS, 0.660 for MRPS27, 0.644 for SLC25A20, and 0.606 for SYNJ2BP (Fig. **7C**). The comprehensive ROC curve analysis of both datasets resulted in AUC values of 0.834 and 0.868, respectively, indicating the model’s high diagnostic value for AD prediction (Fig. **7B**, **D**).

#### 3.4.3 Nomograms for Alzheimer’s Disease Diagnosis

To facilitate AD diagnosis, nomograms incorporating trait genes were developed (Fig. **7E**). In this nomogram, each trait gene is assigned a specific score, and the cumulative score is calculated by summing the scores of all trait genes. This total score reflects varying levels of AD risk. The calibration curves confirmed the nomogram’s accuracy in predicting AD onset (Fig. **7F**).

#### 3.4.4 Analysis of Immune Cell Infiltration

**Fig. (8):**
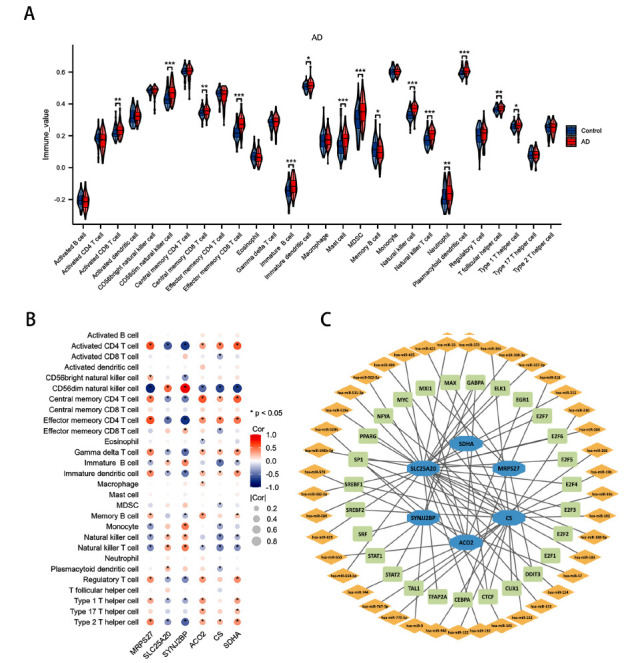
The immune status was analyzed based on six key MRGs, and the relationship between transcription factor, miRNA, and MRGs was explored. (**A**) The violin plot of the immune cell proportions in AD patients; (**B**) The correlation between 6 key MRGs and immune cells; (**C**) The miRNA-MRGs- Cytoscape constructed transcription factor interaction network.

To assess the immune status of AD patients, immune-infiltrating cells were analyzed using CIBERSORT. The violin plot revealed significantly higher levels of CD56dim natural killer cells, effector memory CD8 T cells, mast cells, natural killer cells, and natural killer T cells in AD samples compared to the control group (Fig. **8A**).

#### 3.4.5 Correlation Analysis Between Immune Cell Subsets and Critical MRGs

Intriguingly, among the 28 immune cell types, activated CD4 T cells, central memory CD4 T cells, effector memory CD4 T cells, gamma delta T cells, immature dendritic cells, regulatory T cells, type 1 T helper cells and type 2 T helper cells positively correlated with MRPS27, ACO2, CS, and SDHA but negatively correlated with SLC25A20 and SYNJ2BP. Conversely, CD56dim natural killer cells negatively correlated with MRPS27, ACO2, CS, and SDHA but positively correlated with SLC25A20 and SYNJ2BP. Notably, CD56dim natural killer cells exhibited the strongest correlation with all six key MRGs (Fig. **8B**), suggesting a close interplay between immune cells and critical MRGs.

#### 3.4.6 Construction of the miRNA-MRGs-TF Interaction Network

The miRNA-MRGs-TF interaction network was analyzed and visualized using Cytoscape software, revealing a network composed of 74 nodes and 75 edges (Fig. **8C**). Specifically, six MRGs interacted with 27 transcription factor (TF) genes and 41 microRNAs (miRNAs). SLC25A20, with the highest degree value of 31, was associated with 11 TF genes and 20 miRNAs, followed by CS, SYNJ2BP, ACO2, MRPS27, and SDHA. Among the TFs, GABPA showed the closest association with the critical MRGs.

#### 3.4.7 Construction of the Protein-Drug Interaction Network

Utilizing the drug molecule information and enrichment tool in the DSigDB database, three drug molecules-Congo red, kynurenic acid, and lamotrigine-were selected from the top 15 potential drug molecules based on their *P*-values.

## 4 DISCUSSION

Despite the discovery of Alzheimer’s disease (AD) over a century ago, its underlying pathogenesis and effective treatment remain elusive. The consensus holds that the extracellular accumulation of Amyloid-β (Aβ) peptides represents a defining neuropathological hallmark of AD. Recent advancements suggest that this accumulation can induce mitochondrial dysfunction and fragmentation [4, 16]. Consequently, mitochondrial dysfunction, which constitutes an early event and a common pathological mechanism in AD, plays a pivotal role in the progression of the disease [20, 35]. To this end, we have identified genes related to mitochondrial dysfunction, aiming to discover novel molecular markers that could facilitate early AD diagnosis and enhance clinical drug therapy.

In our study, we downloaded gene expression profiles from the GEO database, specifically GSE132903 and GSE5281, to investigate potential candidate genes for AD. Utilizing the training set GSE132903 for Weighted Gene Co-expression Network Analysis (WGCNA), we selected 724 module genes within the green module for comparison with mitochondria-associated genes listed in the MitoCarta database, yielding 60 Mitochondria-Related Genes (MRGs). We then employed Gene Ontology (GO) terms and Kyoto Encyclopedia of Genes and Genomes (KEGG) pathways to elucidate the biological functions most pertinent to AD.

GO enrichment analysis revealed that mitochondrial gene expression, mitochondrial translation, the inner mitochondrial membrane protein complex, and mitochondrial protein-containing complexes are associated with AD. Notably, both GO terminology and KEGG pathway analysis exhibited a correlation with the Tricarboxylic Acid (TCA) cycle and Oxidative Phosphorylation (OXPHOS). The inner mitochondrial membrane protein complex primarily comprises Electron Transport Chain (ETC) complexes, which, as crucial molecules of OXPHOS, participate in mitochondrial energy metabolism [36]. Most ETC complexes, including Complexes I, III, and IV, are predominantly encoded by mitochondrial genes, indicating that mitochondrial gene expression and translation are determinants in specifying the number of ETC complexes [37]. Deletion of mitochondrial genes directly disrupts the OXPHOS process, resulting in mitochondrial dysfunction [38]. Studies have shown that the absence or inhibition of ETC complexes can trigger the onset and progression of neurodegenerative disorders, such as AD. For instance, oxidative stress arising from Complex I inhibition enhances the production of damaged proteins, leading to neuronal injury [39]. The TCA cycle, a metabolic pathway in mitochondria, plays a central role in ATP synthesis through OXPHOS by providing electron carriers [40]. Neurons heavily rely on these pathways to furnish energy for processes like synaptic transmission [16]. A decrease in the concentration of TCA cycle enzymes and enzyme complexes disrupts the OXPHOS pathway, contributing to the occurrence and progression of AD [41, 42]. These findings are consonant with the results of our enrichment analysis.

In recent years, machine learning has emerged as a powerful tool for diagnosing and screening key genes associated with Alzheimer’s disease (AD), leveraging its capabilities in processing large-scale data, pattern recognition, prediction, and comprehension of the intricate mechanisms underlying AD pathology [43, 44]. This study employed machine learning models-specifically Random Forest (RF), Support Vector Machine (SVM), and Least Absolute Shrinkage and Selection Operator (LASSO)-alongside Protein-Protein Interaction (PPI) network analysis to identify eight pivotal genes with diagnostic significance in Mitochondrial Ribosomal Genes (MRGs), subsequently constructing diagnostic models. After excluding genes without expression differences, six crucial MRGs were ultimately screened: ACO2, CS, MRPS27, SDHA, SLC25A20, and SYNJ2BP. The diagnostic performance of the six key MRGs was assessed using ROC curves, demonstrating their significance. The Receiver Operating Characteristic (ROC) diagnostic model exhibited robust performance across diverse subgroups of AD patients. Notably, diagnostic accuracy varied by disease stage, with early-stage AD outperforming late-stage AD, particularly with MRPS27, SDHA, SLC25A20, and SYNJ2BP demonstrating more consistent performance (Figs. **S1, 2**). Gender-specific analysis revealed gender-dependent differences in molecular performance: among males, ACO2 and SDHA displayed superior diagnostic accuracy, whereas among females, CS, MRPS27, SLC25A20, and SYNJ2BP showed more stable diagnostic performance (Figs. **S3, 4**).

Mitochondrial ribosomal protein S27 (MRPS27) and succinate dehydrogenase complex flavoprotein subunit A (SDHA) are instrumental in the synthesis of Electron Transport Chain (ETC) complexes. MRPS27, a component of mitochondrial ribosomal protein, stimulates mitochondrial mRNA to facilitate the translation of the ETC complex, which is vital for mitochondrial function [45]. Downregulation of MRPS27 results in reduced ETC complexes, thereby accelerating AD progression, aligning with our observations. SDHA, the largest subunit comprising succinate dehydrogenase (Complex II), oxidizes succinic acid to pyruvate in mitochondria, serving as a crucial link in energy metabolism. Autosomal mutations in SDHA have been linked to mitochondrial dysfunction and mitochondrial diseases [46]. The absence of Complex II is intimately associated with the pathology of neurodegenerative disorders [47] (Table **3**).

Solute Carrier Family 25 Member 20 (SLC25A20) is a mitochondrial membrane carrier protein primarily responsible for translocating fatty acids into the mitochondrial matrix, which undergoes oxidation *via* the mitochondrial fatty acid β-oxidation pathway [48]. The liver converts fatty acids into ketone bodies through this pathway and the process of ketogenesis. These ketone bodies serve as metabolic fuel for tissues such as the brain and muscles [49]. Experiments indicate that while overall brain energy metabolism decreases in AD patients, brain ketone metabolism remains normal, suggesting that the AD brain compensates for energy deficiency through ketone bodies [50]. It is hypothesized that SLC25A20 promotes fatty acid transfer in the mitochondrial fatty acid β-oxidation pathway, accelerating fatty acid metabolism and ketone body production, thereby replenishing missing energy in the brain. Our study observed a significant upregulation of SLC25A20 expression in AD patients compared to controls, supporting this hypothesis.

Synaptojanin 2 Binding Protein (SYNJ2BP) is a mitochondrial outer membrane protein functioning as a cell signaling hub [51]. Mitochondrial outer membrane proteins are enriched at the mitochondria-endoplasmic reticulum contact (MERC) site, enabling mitochondria to regulate Ca^2+^ metabolic processes and communicate with the endoplasmic reticulum. In a mouse model of AD, abnormal increases in MERC cause excessive Ca^2+^ influx into mitochondria, inducing apoptosis [52]. Neuronal experiments show that SYNJ2BP overexpression increases MERC, alters neuronal mitochondrial distribution, and leads to significant reduction and damage of axonal mitochondria, which is considered a pathology of AD [51, 53].

Aconitase 2 (ACO2) and Citrate synthase (CS) are two essential enzymes within the Tricarboxylic Acid (TCA) cycle. ACO2, an isomer of the aconitase enzyme in the TCA cycle, catalyzes the reversible conversion of aconitate to citrate in the second step of the cycle, which is crucial for ATP synthesis and the production of various metabolic intermediates. This reaction impacts the overall energy metabolism of the cell. Similarly, CS serves as a crucial rate-limiting enzyme in the TCA cycle, catalyzing the synthesis of citric acid from oxaloacetate and acetyl-CoA, thereby playing a fundamental role in energy production [54, 55]. It marks the entry point for acetyl groups into the TCA cycle. Evidence suggests that impairments in these enzymes may disrupt energy metabolism, potentially exacerbating the neuropathological mechanisms associated with Alzheimer’s disease (AD). A study has shown that ACO2 deficiency causes energy metabolism and mitochondrial dysfunction in neurons, leading to neurological symptoms in patients [54]. Furthermore, reduced CS activity has been observed in AD patients, aligning with our findings [56]. The functions of ACO2 and CS within the TCA cycle likely play significant roles in the energy metabolism abnormalities seen in AD. Moreover, some of the six key mitochondrial dysfunction-related genes (MRGs), including ACO2 and CS, have been implicated in regulating immune responses. For instance, in immune cell studies, it was discovered that nitric oxide produced by macrophages targets ACO2, disrupting the TCA cycle and altering the metabolic pathway of M1 macrophages. This leads to the inhibition and loss of the electron transport chain (ETC) complex, affecting macrophage activity. However, the roles of other essential molecules on the immune system and their potential impacts on AD pathology through this pathway remain to be further explored. In summary, ACO2 and CS are vital enzymes in the TCA cycle that play crucial roles in energy metabolism and are implicated in the neuropathological mechanisms of AD. Additionally, some of these key MRGs have been shown to regulate immune responses, suggesting potential new avenues for research into AD pathology and treatment.

Numerous studies have demonstrated that neuroinflammation plays a pivotal role in the pathophysiology of Alzheimer’s disease (AD) [57]. In particular, recent evidence has shown that neuroinflammation in AD is not merely a reactive response to the presence of senile plaques and neurofibrillary tangles; rather, it actively participates in their formation and development, potentially exerting an equal or even greater influence on their pathogenesis [58]. Our findings are consistent with previous studies reporting an upregulation of activated CD56dim natural killer (NK) cells, effector memory CD8 T cells, mast cells, conventional NK cells, NKT cells, and neutrophils [44, 59]. Distinct immune cell subsets play crucial roles in AD. The harmful mediators released by mast cells can exacerbate neuroinflammation and contribute to poor prognostic outcomes in neurodegenerative diseases [60]. Similarly, neutrophils encapsulate Aβ plaques through neutrophil extracellular traps, leading to excessive Aβ accumulation and heightened neuroinflammation [61, 62].

This study has identified significant correlations between critical MRGs and CD56dim NK cells. CD56dim NK cells, a subset of human NK cells, release inflammatory cytokines and stimulate other immune cells [63], thereby participating in the initiation and progression of brain inflammation [64]. In AD patients, circulating NK cells infiltrate the brain and promote an inflammatory response [65]. Studies have indicated that reducing NK cells can markedly decrease neuroinflammation, promote neurogenesis, and ameliorate cognitive impairment [66]. Our results show that specific immune cell types, particularly NK cells and T cells, exhibit notable correlations with MRG expression in the context of AD pathology, aligning with previous research findings.

Through the miRNA-MRGs-transcription factor interaction network, we found that GA Binding Protein Transcription Factor Subunit Alpha (GABPA) was most closely correlated with key MRGs. GABPA encodes the Nuclear Respiratory Factor 2 subunit, which is crucial for mitochondrial biogenesis and energy production [67-70]. Similarly, GABPA controls the production of ETC complexes, which in turn influences ATP synthesis. Experiments have shown that mitochondrial dysfunction resulting from GABPA disruption promotes early embryonic lethality in mice [71]. Notably, the role of peroxisome proliferator-activated receptor gamma (PPARG) in regulating mitochondria and its implications in AD cannot be overlooked. PPARG facilitates mitochondrial function recovery by activating genes related to mitochondrial biogenesis, regulates mitochondrial energy metabolism to enhance ATP production [72], and activates the expression of antioxidant enzymes to mitigate oxidative stress damage. These effects are intimately tied to the pathogenesis and progression of AD, aiding in the improvement of neuronal cell energy status and the delay of disease progression [73].

Through the interaction network of miRNAs, microRNA response elements (MRGs), and transcription factors, we identified GABPA as the most significantly correlated with pivotal MRGs. Specifically, GA Binding Protein Transcription Factor Subunit Alpha (GABPA) encodes the subunit of Nuclear Respiratory Factor 2, a crucial factor in mitochondrial biogenesis and energy metabolism [70]. Analogously, GABPA regulates the production of electron transport chain (ETC) complexes, which in turn impacts ATP generation. Experimental evidence suggests that mitochondrial dysfunction resulting from GABPA disruption accelerates early embryonic lethality in mice [71]. Notably, the role of PPARG in mitochondrial regulation and its implications in Alzheimer’s Disease (AD) cannot be overlooked. PPARG (Peroxisome Proliferator-Activated Receptor Gamma) facilitates mitochondrial functional recovery by activating genes related to mitochondrial biogenesis, modulates mitochondrial energy metabolism to augment ATP production [72], and activates antioxidant enzymes and antioxidants to mitigate oxidative stress damage. These effects are intricately linked to the pathogenesis and progression of AD, aiding in the enhancement of neuronal cell energy status and the delay of disease progression [73].

Furthermore, we constructed a protein-drug interaction network to provide clinical insights into drugs for AD treatment and prognosis. A total of three drugs were screened: Congo red, kynurenic acid, and lamotrigine. Congo red, a fiber-specific dye, binds to amyloid, inhibiting amyloid formation and ion channel function in laboratory animals, thereby reducing Aβ neurotoxicity, as demonstrated in early AD studies [74, 75]. Kynurenic acid (KYNA), a key compound in the kynurenine pathway, competitively inhibits excitatory amino acid receptors, protecting nerves from the neurotoxicity of its ligand [76]. However, elevated KYNA levels in schizophrenia patients can lead to cognitive impairment [77], indicating KYNA’s complex dual effects on the nervous system. Thus, when developing KYNA as a therapeutic, its potential side effects and safety must be thoroughly considered. Future research should focus on elucidating KYNA’s dual mechanisms, exploring dose optimization and combination therapies to enhance neuroprotective effects while minimizing adverse impacts. Lamotrigine (LTG), commonly used to treat epilepsy, reduces glutamate release by inhibiting neuronal sodium channels [78]. LTG has been reported to ameliorate AD pathology through multiple mechanisms, including alleviating brain inflammatory responses and reducing Aβ levels in AD mice [79]. These findings provide compelling evidence for LTG’s feasibility as an AD therapeutic. However, like any drug, LTG has potential side effects such as skin rash, dizziness, and drowsiness, and long-term use may cause toxicity to other organs [80]. Therefore, further clinical research and safety assessments are warranted before LTG’s application in AD treatment.

Moreover, recent groundbreaking research has revealed that iron metabolism may influence AD progression by modulating mitochondrial function. Age-related iron deposition in various brain regions can impair normal cognitive functions and behaviors [81]. Imbalances in iron metabolism lead to mitochondrial iron overload, where excess iron catalyzes the Fenton reaction, generating excessive reactive oxygen species (ROS), inducing oxidative stress that further damages functionally defective mitochondrial complexes. This results in energy deficiencies and triggers a cascade of apoptotic events, ultimately leading to neuronal apoptosis and the onset of neurodegenerative diseases, such as AD [82]. Additionally, iron imbalance and oxidative stress, either collaboratively or independently, promote excessive amyloid-β (Aβ) production by activating β- or γ-secretases and inhibiting α-secretase. They also induce tau protein hyperphosphorylation by activating protein kinases such as glycogen synthase kinase-3β (GSK-3β) and cyclin-dependent kinase 5 (CDK5) while inhibiting protein phosphatase 2A (PP2A) [83]. These alterations caused by iron ion imbalance exacerbate iron distribution and deposition, creating a vicious cycle that may ultimately facilitate AD development.

Previous studies have recognized a non-negligible link between AD and mitochondrial structure and function. The study of the related molecular mechanisms aids in exploring AD molecular markers and provides an auxiliary scheme for clinical treatment. Researchers have explored single factors such as mitochondrial quality control systems, but studies on mitochondria-dysfunction-related molecules causing AD still need further investigation. This paper employed various bioinformatics and machine-learning methods to identify mitochondria-associated genes. Our model demonstrates high accuracy and stability across independent test sets, and the prediction of targeted drugs offers new possibilities for AD treatment. However, our study has limitations: (1) Some biomarkers related to mitochondrial dysfunction require further literature support; (2) The therapeutic significance of the critical MRGs obtained through AD screening still needs further clinical validation; (3) The functional information of some genes is limited, necessitating model improvements with future updates. Future research can address these limitations by conducting functional validation studies and exploring the clinical relevance of biomarkers across different populations, thereby enhancing the clinical applicability of our models. We recommend incorporating more diverse population samples and other neurodegenerative diseases into future versions of the diagnostic model to assess its generalization ability and applicability across various contexts.

## CONCLUSION

In summary, this study used various bioinformatics methods to identify the role of six key MRGs in AD and built a diagnostic model based on these genes. We revealed the correlation of MRGs with infiltrating immune cells and searched for AD-targeting compounds. Our study provides new insights into the exploration of novel biomarkers and targeted drugs for AD.

## Figures and Tables

**Fig. (1):**
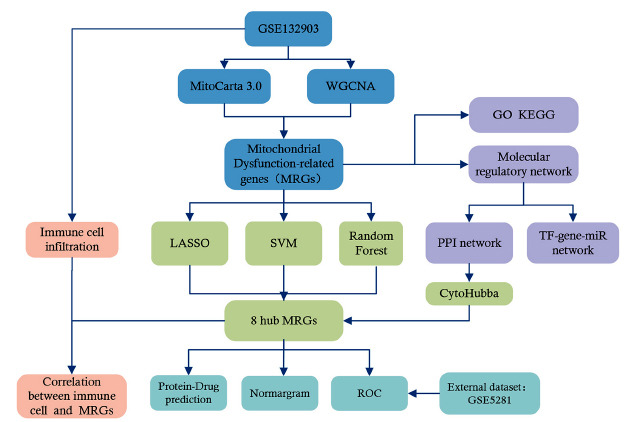
Flow chart of the study.

**Table 1:** Details of the obtained data set.

**Dataset**	**Platform**	**Control**	**IS**	**Author**	**Country**	**Submission**	**Sample**	**Application**
GSE132903	GPL10558	98	97	Piras IS	USA	2010	brain tissue section	Identification
GSE5281	GPL570	74	87	Stephan DA	USA	2014	brain tissue section	Validation

**Table 2:** Information on the 6 key mitochondrial dysfunction-related genes.

**Gene**	**Full name**
MRPS27	Mitochondrial Ribosomal Protein S27
SLC25A20	Solute Carrier Family 25 Member 20
SYNJ2BP	Synaptojanin 2 Binding Protein
ACO2	Aconitase 2
CS	Citrate Synthase
SDHA	Succinate Dehydrogenase Complex Flavoprotein

**Table 3:** 3 AD targeted therapeutic drugs screened from protein-drug interaction network.

**Name**	**Chemical Formula**	***P*-value**	**Structure**	**Genes**
Congo red	C_32_H_22_N_6_Na_2_O_6_S_2_	0.00499	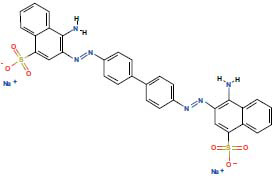	SLC25A20
Kynurenic acid	C_10_H_7_NO_3_	0.005239	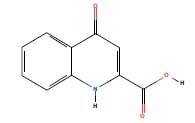	SLC25A20
Lamotrigine	C_9_H_7_Cl_2_N_5_	0.005488	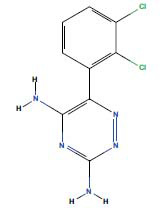	SLC25A20

## Data Availability

The data supporting this study’s findings are available from the corresponding author upon reasonable request.

## LIST OF ABBREVIATIONS

AD: Alzheimer’s Disease
MRGs: Mitochondrial Dysfunction-Related Genes
NFTs: Neurofibrillary Tangles
Aβ: Amyloid-β
ATP: Adenosine Triphosphate
GEO: Gene Expression Omnibus
WGCNA: Weighted Gene Co-expression Network Analysis
GO: Gene Ontology
BP: Biological Process
CC: Cellular Component
MF: Molecular Function
KEGG: Kyoto Encyclopedia of Genes and Genomes
RF: Random Forest
LASSO: Least Absolute Shrinkage and Selection Operator
SVM: Support Vector Machine
PPI: Protein-protein Interaction
TF: Transcription Factor
ROC: Receiver Operating Characteristic
TCA: Tricarboxylic Acid
OXPHOS: Oxidative Phosphorylation
ETC: Electron Transport Chain
NK: Natural Killer
MSigDB: Molecular Signature Database
KYNA: Kynurenic Acid
LTG: Lamotrigine
MERC: Mitochondria-endoplasmic Reticulum Contact
