# Supplementary material for: Development of a Novel Mitochondrial Dysfunction-Related Alzheimer’s Disease Diagnostic Model Using Bioinformatics and Machine Learning
Source: Curr Alzheimer Res. 2024 Dec 26;22(1):19–37. doi: 10.2174/0115672050353736241218054012 (PMC12376136; doi:10.2174/0115672050353736241218054012)
Supplement: Supplementary file 1 [file CAR-22-1-19_SD1.pdf]

# Supplementary Materials

# Development of a Novel Mitochondrial Dysfunction-Related Alzheimer's Disease Diagnostic Model Using Bioinformatics and Machine Learning

Kuo Zhang[1], Kai Yang[2], Gongchang Yu[1,*] and Bin Shi[1,*]

*[1]Neck-Pain Hospital of Shoulder and Lumbocrural Shandong First Medical University, Shandong First Medical University & Shandong Academy of Medical Sciences, Jinan, China; [2]Shandong University of Traditional Chinese Medicine, Department of Neurology, Jinan, China*

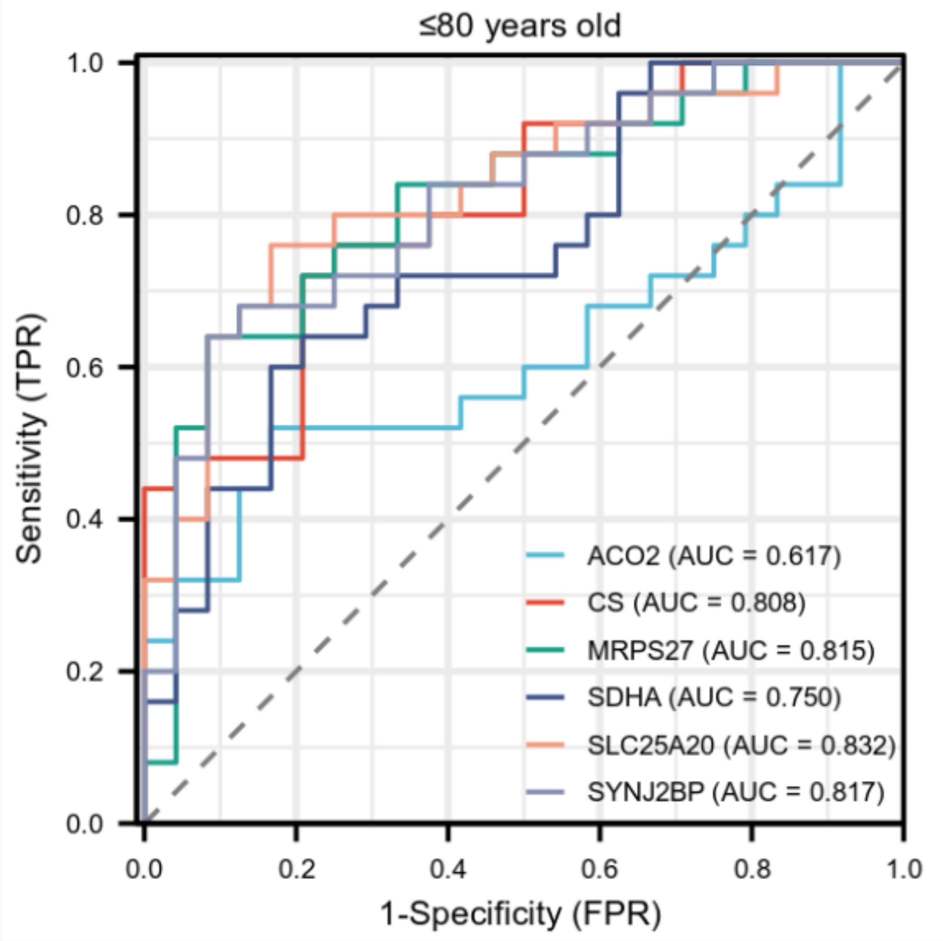


**Fig. S1.**

1875-5828/25 © 2025 Bentham Science Publishers

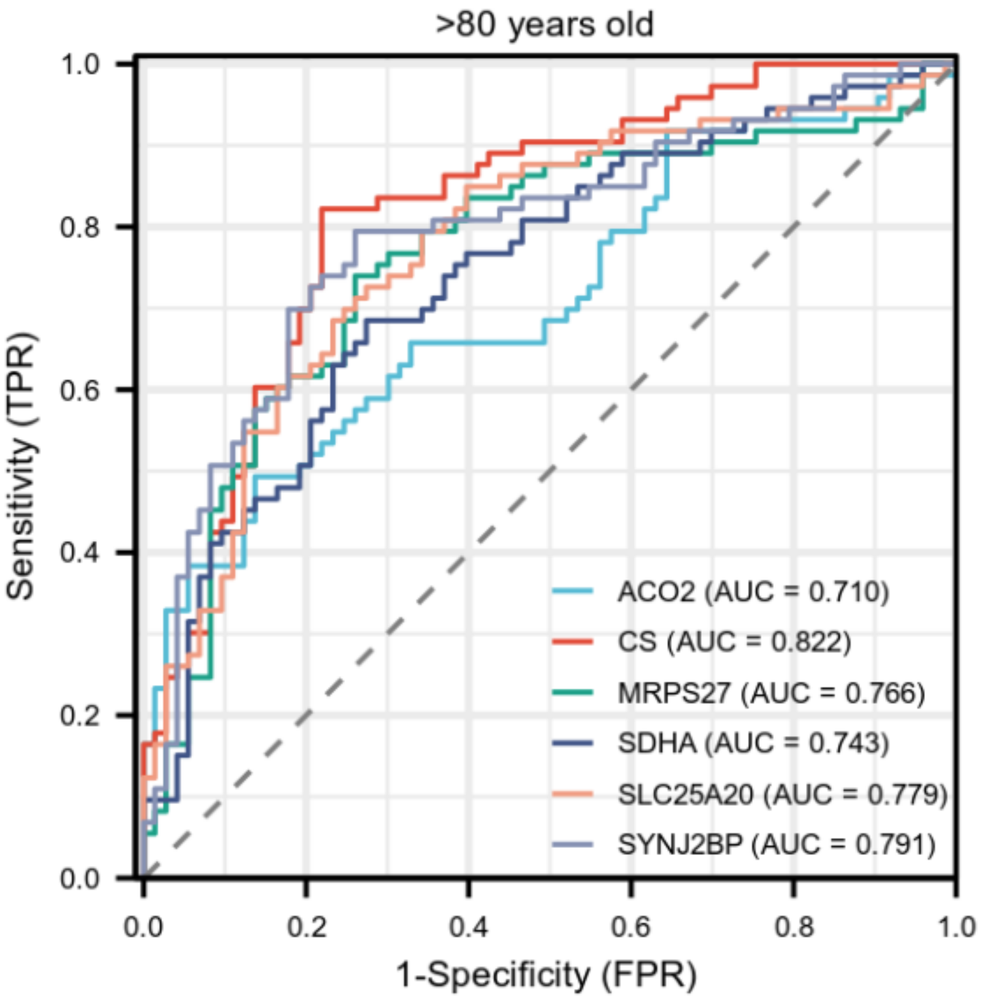
>80 years old
1.0
0.8
0.6
0.4
0.2
0.0
Sensitivity (TPR)
0.0
0.2
0.4
0.6
0.8
1.0
1-Specificity (FPR)
ACO2 (AUC = 0.710)
CS (AUC = 0.822)
MRPS27 (AUC = 0.766)
SDHA (AUC = 0.743)
SLC25A20 (AUC = 0.779)
SYNJ2BP (AUC = 0.791)

**Fig. S2.**

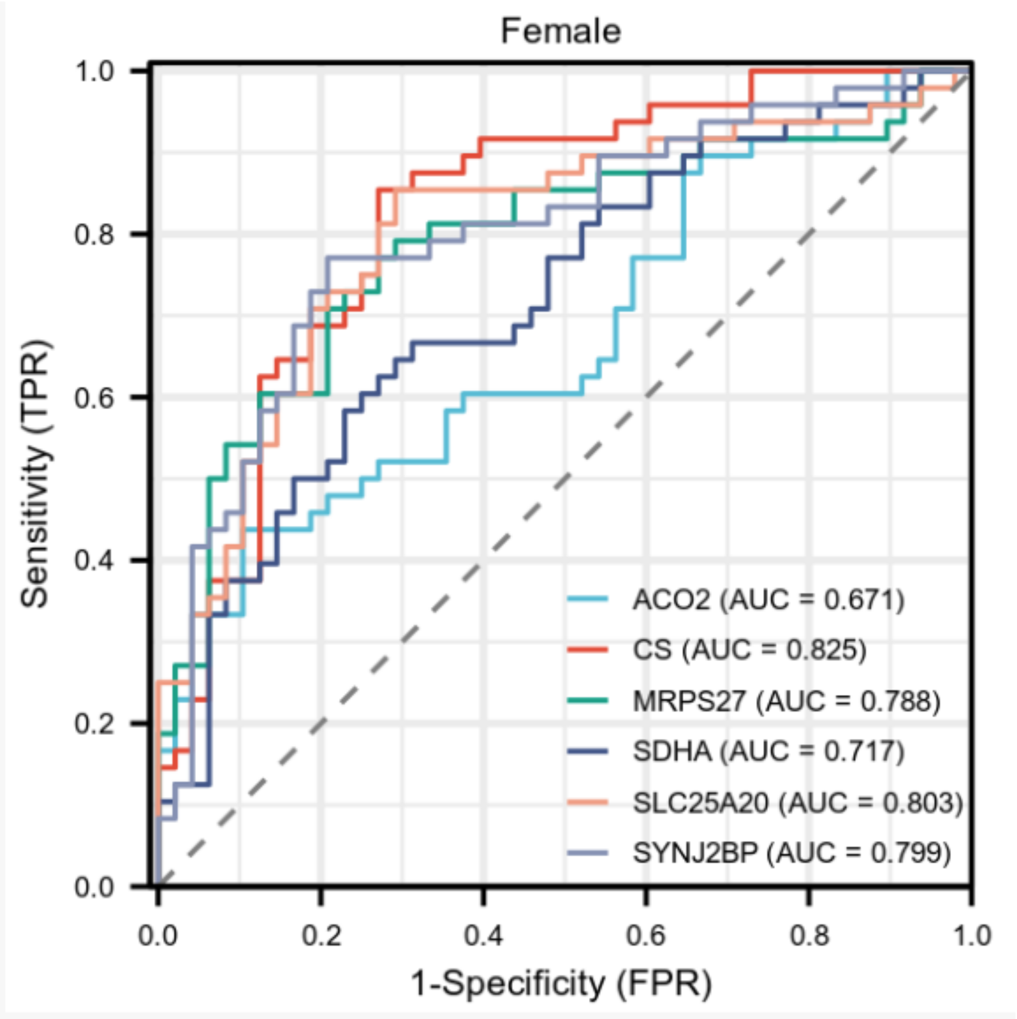
Female
1.0
0.8
0.6
0.4
0.2
0.0
Sensitivity (TPR)
0.0
0.2
0.4
0.6
0.8
1.0
1-Specificity (FPR)
ACO2 (AUC = 0.671)
CS (AUC = 0.825)
MRPS27 (AUC = 0.788)
SDHA (AUC = 0.717)
SLC25A20 (AUC = 0.803)
SYNJ2BP (AUC = 0.799)

**Fig. S3.**

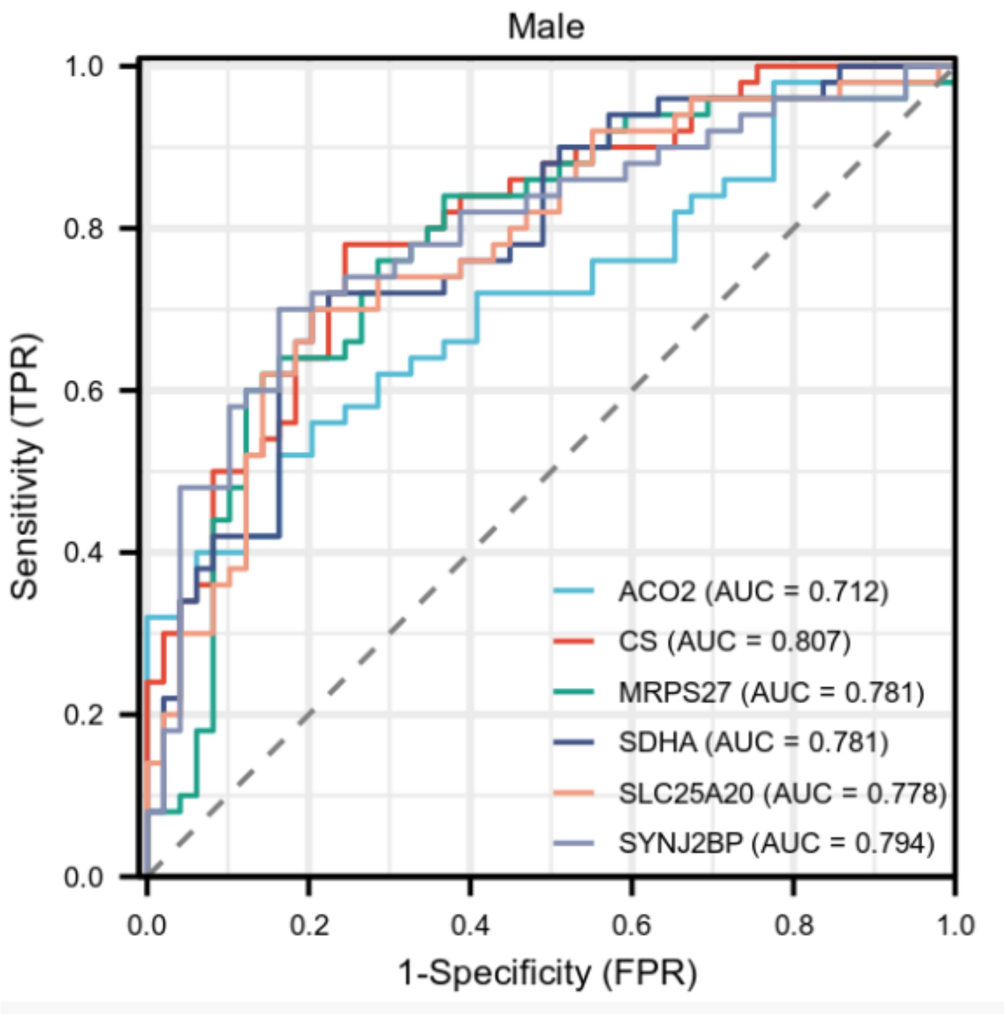
Male
1.0
0.8
0.6
0.4
0.2
0.0
Sensitivity (TPR)
0.0
0.2
0.4
0.6
0.8
1.0
1-Specificity (FPR)
ACO2 (AUC = 0.712)
CS (AUC = 0.807)
MRPS27 (AUC = 0.781)
SDHA (AUC = 0.781)
SLC25A20 (AUC = 0.778)
SYNJ2BP (AUC = 0.794)

**Fig. S4.**
